# Structural Based Screening of Antiandrogen Targeting Activation Function-2 Binding Site

**DOI:** 10.3389/fphar.2018.01419

**Published:** 2018-11-30

**Authors:** Yangguang Liu, Meng Wu, Tianqi Wang, Yongli Xie, Xiangling Cui, Liujun He, Yang He, Xiaoyu Li, Mingliang Liu, Laixing Hu, Shan Cen, Jinming Zhou

**Affiliations:** ^1^Key Laboratory of the Ministry of Education for Advanced Catalysis Materials, Department of Chemistry, Zhejiang Normal University, Jinhua, China; ^2^Institute of Medicinal Biotechnology, Chinese Academy of Medical Sciences, Beijing, China

**Keywords:** androgen receptor, structural based drug design, antiandrogen, castration-resistant prostate cancer, activation function-2

## Abstract

Androgen receptor (AR) plays a critical role in the development and progression of prostate cancer (PCa). Current antiandrogen therapies induce resistant mutations at the hormone binding pocket (HBP) that convert the activity of these agents from antagonist to agonist. Thus, there is a high unmet medical need for the development of novel antiandrogens which circumvent mutation-based resistance. Herein, through the analysis of AR structures with ligands binding to the activation function-2 (AF2) site, we built a combined pharmacophore model. *In silico* screening and the subsequent biological evaluation lead to the discovery of the novel lead compound IMB-A6 that binds to the AF2 site, which inhibits the activity of either wild-type (WT) or resistance mutated ARs. Our work demonstrates structure-based drug design is an efficient strategy to discover new antiandrogens, and provides a new class of small molecular antiandrogens for the development of novel treatment agents against PCa.

## Introduction

Prostate cancer (PCa) is one of the most common cancers and the second leading cause of cancer death in men in the western countries (Torre et al., 2015). Androgen receptor (AR), a member of nuclear receptor family that is activated by binding of androgens (Roy et al., 1999), plays an important role in promoting the development of PCa (Dong et al., 2005). The proliferation and survival of PCa cells is critically dependent on the AR signaling axis. Moreover, as the disease evolves from androgen-sensitive cancer to castration-resistant prostate cancer (CRPC), the growth of cancer cells still rely on AR signaling axis (Jenster, 1999; Taplin, 2007). Thus, AR has become the most important therapeutic target for the treatment of PCa (Aragon-Ching, 2014; Carver, 2014; Culig, 2014).

Androgen receptor antagonist prevents androgens from carrying out their biological activity by directly binding and blocking the AR ligand binding domain (LBD), or by inducing repressive activity (Maeda and Usami, 2002; Gillatt, 2006) like blocking the nuclear translocation of AR, dismissing AR binding to DNA, thus inhibiting the androgen induced transcriptional activation of AR (Maeda and Usami, 2002; Gillatt, 2006). Current clinically used antiandrogens such as flutamide (FLU) (Goldspiel and Kohler, 1990), bicalutamide (BIC) (Blackledge et al., 1997), and recently approved enzalutamide (ENZ) (Semenas et al., 2013) mainly target the hormone binding pocket (HBP) of the AR LBD. These approved antiandrogens have greatly improved the survival and life quality of the PCa patients. However, after the initially effective response, most tumors progress to CRPC under the treatment of antiandrogens, and no curative therapy is available up to now. It has been widely accepted that the acquired AR mutation is an important cause leading to the drug resistance of PCa toward antiandrogens. For examples, T877A mutant would turn FLU into AR agonist (Bohl et al., 2005b), and W741C would turn BIC into AR agonist (Bohl et al., 2005a). Recently, a novel F876L mutant which turned ENZ and ARN509 from AR antagonist to AR agonist was identified in preclinical models and in the patients being treated with ARN509 (Joseph et al., 2013). Therefore, the development of novel antiandrogens to circumvent the mutation induced resistance is highly demanded.

Besides of HBP, several other binding sites on AR like activation function-2 (AF2), binding function-3 (BF3), the DNA binding site, and activation function-1 (AF1) have been attracted attentions in novel antiandrogen development (Haendler and Cleve, 2012). Among these binding sites, the AF2 site, which forms through the agonistic conformational change of helix-12 upon the binding of agonist (hormone) to the HBP site, plays a critical role in the activation of AR. AF2 is essential for the recruitment of various AR coactivators. Molecules that bind to AF2 will block the interaction of AR with coactivators by a competitive mechanism, thus inhibiting the transcriptional activity of AR. Such molecules represent a novel type of antiandrogens other than the traditional antiandrogens which bind HBP. To date, several ligands have been identified to bind to the AF2 site, and exhibit potent activities in antagonizing the AR signaling, which inhibit the proliferation of AR dependent PCa cells (Andersen et al., 2010; Axerio-Cilies et al., 2011; Ravindranathan et al., 2013; Li et al., 2014; Munuganti et al., 2014).

Up to now, more than 100 crystal structures of AR-LBD have been deposited in Protein Database Bank (PDB^1^). Structure-based drug design (SBDD) has emerged as an effective way to obtain lead compounds targeting the AF2 site (Axerio-Cilies et al., 2011; Blanco et al., 2012; Caboni et al., 2012). The AF2 site is located at a hydrophobic groove flanked with regions of positive and negative charges, “charge clamps” constituted by parts of helix-3, helix-5 and helix-12 (Hur et al., 2004). Several structures with ligand binding to the AF2 site have been released, and these structures may provide valuable information for SBDD in discovery of ligands targeting the AF2 site. Through structural analysis of these complexes, we found the ligands binding to the AF2 site adopted two quite different binding modes. Therefore, we built a pharmacophore model via combining these two binding modes. *In silico* screen and the subsequent biological evaluation leads to the discovery of the novel lead compound IMB-A6 that binds to the AF2 site. Our work demonstrates that SBDD is an efficient strategy in the development of antiandrogens, and provides a new class of non-peptidic, small molecule AR/coactivator selective blockers for the development of novel treatment agents of PCa.

## Materials and Methods

### *In silico* Screening

The AR structures with ligands binding to the AF2 site were downloaded from PDB^1^ database and were further superimposed in the molecular modeling software discovery studio (DS) based on the sequence alignment. The pharmacophore model was generated by pharmacophore module in DS. The pharmacophore based screening was performed through Screen Library protocol in Pharmacophore module of DS. The hits after pharmacophore filtration was screened using the docking module LIBDOCK in DS (Diller and Merz, 2001) against the AF2 binding site on AR structure (PDB-ID:1T7R), which was selected as the target structure for the molecular docking because the structure had the highest resolution (1.4 Å) and had a FxxLF peptide motif binding to AF2 site. The outputted results was further evaluated using the docking module CDOCKER in DS. The ligand binding region was defined as a sphere of 12 Å radius around the binding site. The final candidates with top 200 scores were then visually inspected to check the binding mode. At the end, 12 compounds were selected and purchased from Mule company.

Molecular similarity search based on IMB-A6 structure was performed using the structural similarity query section in Zinc database website. The compounds with tanimoto coefficient more than 70% were found out and the structures were deposited into a database. These compounds were further screened through CDOCKER in DS. After visual inspection, the candidate compounds were finally purchased for the further bioassay.

### Cell Lines

The LNCaP cell line was purchased from American Type Culture Collection (ATCC, CRL-1740), and cultured with RPMI-1640 supplemented with 10% Fetal Bovine Serum (GiBCO). The PC-3 cell line was kindly provided by Dr J. H. Wu (McGill University, Canada), and cultured with F-12K supplemented with 10% Fetal Bovine Serum.

### MTT Assay

LNCaP cells were seeded at a density of 4–5 × 10^3^ cells per well in 96-well plate in complete RPMI-1640 growth medium, or PC-3 cells in complete F-12K growth medium at the same density. After overnight incubation, 1 μL dimethyl sulfoxide (DMSO, Sigma-Aldrich) or 1 μL test compounds were added to each well at the designated concentration. After 72 h incubation (37°C, 5% CO_2_), 20 μL of MTT (Invitrogen) solution (5 mg/mL in PBS) were added per well and incubated for another 2 h (37°C, 5% CO_2_). The MTT formazan formed by metabolically viable cells was dissolved in 100 μL isopropanol. The absorbance was measured at 570 nm wavelength on a plate reader (EnSpire 2300, PerkinElmer). Experiments were performed in triplicate. The value of DMSO group was defined as 100%.

### Dual-Luciferase Reporter Assay

Plasmid F876L is full length cDNA of mutant AR that harbors F876L mutation. Plasmid T877A is full length cDNA of mutant AR that harbors T877A mutation. Plasmid W741C+T877A is full length cDNA of mutant AR that harbors W741C and T877A mutations. The F876L and W741C+T877A plasmids were kindly provided by Dr J.H. Wu (McGill University, Canada), and the plasmid T877A was constructed in house. For the reporter assays, 24 h before transfection, PC-3 cells were seeded at a density of 6–7 × 10^4^ cells per well in 24-well plate and subsequently co-transfected with 100 ng of PSA-luc, 20 ng of F876L (20 ng T877A or 50 ng W741C+T877A), and 1 ng of Renilla plasmids using Lipofectamine 2000 reagent (Invitrogen) following the manufacturer’s protocol. 24 h after transfection, the medium was changed to phenol red-free RPMI-1640 supplemented with 10% charcoal-stripped FBS, containing 1 nM of dihydrotestosterone (DHT) 1 μL and 1 μL test compounds at the designated concentration. After further 24 h, the cells were lysed in 100 μL per well passive lysis buffer, and 20 μL of the cell lysates was used for detection of the luciferase activity using Dual Luciferase Assay System (Promega) on a plate reader (Centro XS3 LB 960, Berthold). All experiments were run in triplicate.

### Western Blot

LNCaP cells were seeded at a density of 3 × 10^5^ cells per well in 6-well plate. After overnight incubation, 1 μL DMSO (Sigma-Aldrich) or 1 μL compounds were added to each well at the designated concentration. After another 24 h incubation, the cells were lysed with RIPA. Then the protein lysis buffer was treated with 10% SDS–PAGE for western blot analysis. The following antibodies were used for the detection of proteins: rabbit anti-AR (N-20, 1:500, Santa Cruz), goat anti-PSA (SC-7638, 1:100, Santa Cruz), Mouse anti-actin (1:5000, Abcam) was used as a loading control. Proteins were visualized using anti-mouse or anti-rabbit HRP-conjugated secondary antibodies (1:5000, Santa Cruz) and ECL-Plus (Millipore).

### Immunofluorescence

LNCaP cells were grown on the glass cell culture dish in phenol red-free RPMI-1640 medium containing 10% charcoal-stripped FBS for 24 h, and were then treated with DMSO, 1 nM DHT, 500 nM enzalutamide and 1 nM DHT, 5 μM IMB-A6 and 1 nM DHT, respectively, for further 24 h. Cells were fixed with 4% (vol/vol) paraformaldehyde and permeabilized with 0.2% Triton X-100. Then the cells were incubated with AR antibodies (N-20, 1:100, Santa Cruz). The secondary antibodies, goat anti-rabbit with FITC (Santa Cruz) at 1:100 were used. The counterstain DAPI was used to visualize cell nucleus. The images were detected under UltraVIEW vox spinning disk confocal scanning system (Perkin Elmer) on an Olympus IX81 microscope.

### The Binding Assay of AR HBP

The binding assay of IMB-A6 to AR-LBD protein (HBP site) *in vitro*, relative to dihydro-testosterone (3.6 nM Fluormone DHT Green at final concentration), was evaluated by AR fluorescence polarization (FP) assay, using Polar-Screen AR competitor assay kit (P3018, Invitrogen) on a plate reader (VICTOR X5, PerkinElmer).

### Octet Binding

The Octet RED (FortéBio, Inc., CA, United States), equipped with super streptavidin biosensor chips (FortéBio), was used for the analysis of small molecule protein interactions in fluidics free system. The binding of IMB-A6 to the AR-LBD (A15675^∗^, Invitrogen) was performed by FortéBio Inc. Company ^2^. The purified AR was biotinylated (EZ-Link^®^NHS-Biotin Reagents, Cat. # 21343, Thermo) by 3:1, and then incubated for 1 h at room temperature. Superstreptavidin biosensors (FortéBio Inc., Menlo Park, CA, United States) were coated in a solution containing 1 μM of biotinylated protein for 1 h at room temperature. Duplicated set of sensors were incubated in an assay buffer with 5% DMSO without protein for use as a background binding control. Both sets of sensors were blocked with a solution of 10 mg/ml biocytin for 5 min at room temperature. A negative control of 5% DMSO was also used. Binding of samples to coated and uncoated reference sensors was measured over 120 s. Data analysis on the FortéBio Octet RED instrument was performed using a double reference subtraction (sample and sensor references) in the FortéBio data analysis software.

### Co-immunoprecipitation

For AR and PELP-1 IP assay, LNCaP cells were seeded in 10 cm dishes. 48 h later, the cells were treated with DMSO or 1 μM, 10 μM IMB-A6 for 24 h. The cells were collected and lysed in NP-40-containing buffer for 30 min on ice. Cell lysates were centrifugated at 10,000 ×*g* for 10 min at 4°C and supernatant was transferred to a fresh 1.5 mL tube on ice. The cell lysates were incubated with anti-AR rabbit antibody for 3 h at 4°C. Thirty microliters of protein A-Agarose (Santa Cruz) was added and incubated overnight at 4°C. The beads were washed four times with lysis buffer (cold) and at last boiled in 40 μL sample buffer for 5–10 min. The samples were analyzed by western blotting.

### Statistical Analysis

Unpaired two-tailed Student’s tests or one-way analysis of variance with the Tukey post test were conducted to compare results obtained from the different experimental groups. *P*-value of <0.05 was considered statistically significant. In all figures, error bars indicate the standard deviations for three independent samples. One asterisk (^∗^) indicate a *P*-value lower than 0.05, and two asterisks (^∗∗^) indicate a *P*-value lower than 0.01. All the experiments were repeated more than two times.

## Results

### *In silico* Screening

**FIGURE 1:**
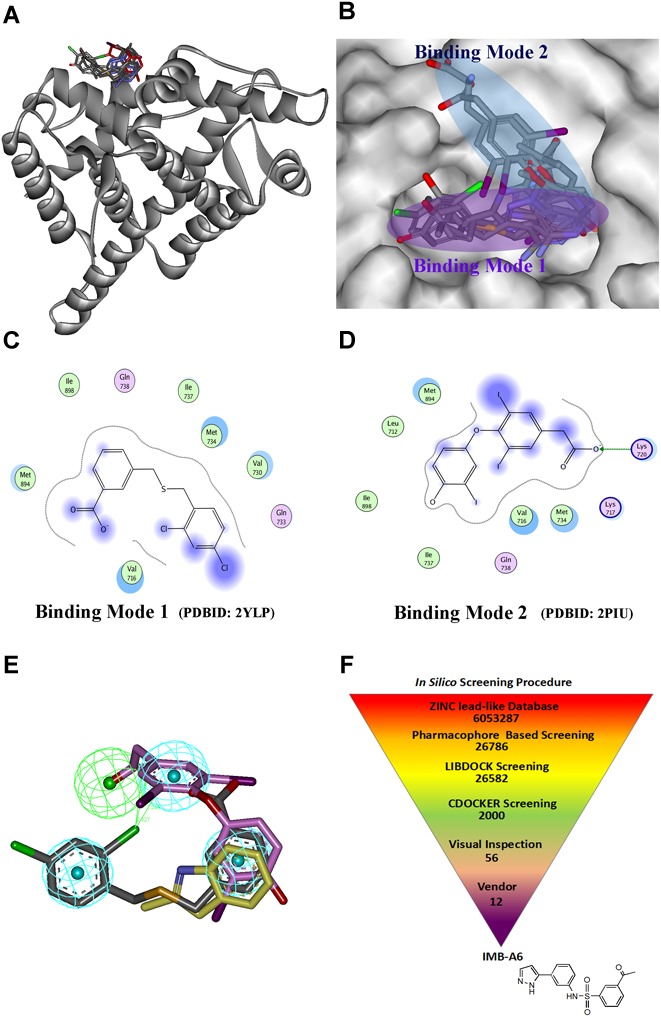
The structural virtual screen targeting the AF2 site of AR. **(A)** The alignment of the structures with ligand binding to AF2 site. **(B)** The ligands binding to the AF2 site adopt two different binding modes. **(C)** The representative for the binding mode 1. **(D)** The representative for the binding mode 2. **(E)** The generating combining binding mode. **(F)** *In silico* screening procedure.

Structure-based drug design has becoming an effective way to discover the lead compound. We herein planned to design the novel antiandrogens that bind to AF2 site using SBDD strategy. During structural analysis of the AR-LBD, we collected the structures with ligands binding to the AF2 site, and the seven structures were found and listed in Supplementary Table S1. A superimposition of these structures through the sequence alignment of the core structures (699–882) of LBD (Figure 1A) identified that the ligands bind to the AF2 site in two quite different binding modes (Figure 1B). In binding mode 1 (derived from PDB-ID: 2YLP), the ligand forms hydrophobic interactions with Val716, Val730, Met734, Ile737, Met894, and Lie898 (Figure 1C). In binding mode 2 (derived from PDB-ID:2PIU), the ligand forms hydrophobic interactions with Leu712, Val716, Met734, Ile737, Met894, and Lie898 as well as forms hydrogen bond interaction with Lys720 (Figure 1D). Importantly, the two binding modes were overlapped at the position of a terminal aromatic ring of each (Figure 1E), and thus that a pharmacophore model was generated in DS, which combined the two binding modes through merging the overlapped aromatic rings. The pharmacophore included three hydrophobic and one hydrogen bond acceptor pharmacophore features (Figure 1E). Such a pharmacophore model included the information of both binding modes, thus becoming a very useful model for the initial screening to improve the screening efficiency.

The schedule of virtual screen was shown in Figure 1F. The purchasable lead-like compounds (∼6 million) from the ZINC database was selected as the ligand database for our virtual screening. First, the database was screened through the generated pharmacophore model. 26786 entries were left, which matched more than three features of the filtering model. The hits after the pharmacophore filtration were screened against the AF2 binding site on AR (PDB-ID:1T7R) using the docking module LIBDOCK in DS (Diller and Merz, 2001), and were further evaluated using the docking module CDOCKER. Finally, after the visual inspection, 12 hits (Supplementary Table S2) were selected and purchased for further assessment through MTT-based cytotoxicity assay and dual-luciferase assay of AR activity.

### Candidate Compounds Evaluation

**FIGURE 2:**
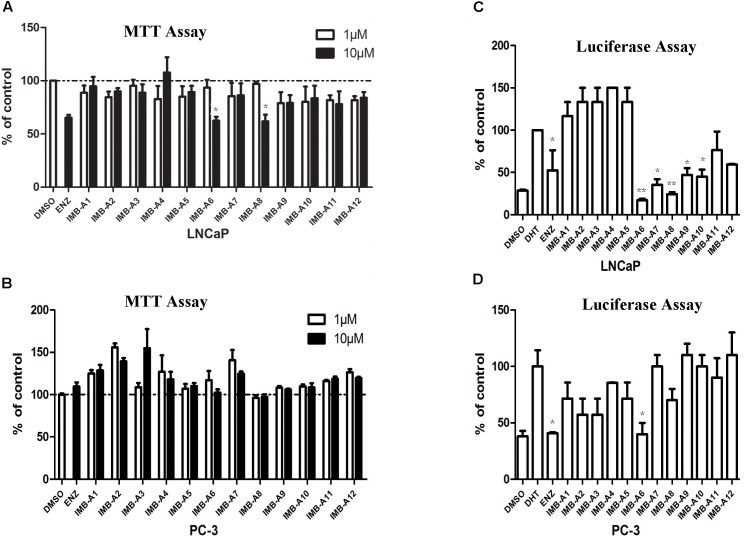
The biological evaluation of candidate compounds. **(A)** MTT assay of candidate compounds in the LNCaP cells. **(B)** MTT assay of candidate compounds in the PC-3 cells. **(C)** The dual-luciferase assay of AR activity for candidate compounds in LNCaP cell. **(D)** The dual-luciferase assay of AR activity for candidate compounds in LNCaP cell. Experiments were in triplicate. All results are shown as mean ± s.d. ^∗^*P* < 0.05, ^∗∗^*P* < 0.01 vs. DMSO group (MTT assay) or DHT group (dual-luciferase assay). ENZ, enzalutamide; DHT, dihydrotestosterone.

To evaluate the biological activity of the 12 hits, we assessed their activity through MTT assay and dual-luciferase assay (Figure 2). In MTT assay, both LNCaP (AR positive) and PC-3 (AR negative) cell lines were used to evaluate the anti-PCa activity of the compounds. 10 μM ENZ treated group was set as the positive control. As a result, compounds IMB-A6 and IMB-A8 inhibited the growth of the LNCaP cells at the concentration of 10 μM, which were comparable to ENZ treated group (Figure 2A). Importantly, both IMB-A6 and IMB-A8 showed no inhibitory activity for the growth of the PC-3 cells, compared to DMSO treated group (Figure 2B). Such difference in cell growth inhibition between LNCaP cells and PC-3 cells suggested the selective AR inhibition activity of IMB-A6 and IMB-A8. AR downstream gene prostate-specific antigen (PSA) is the most commonly used marker to evaluate the transcriptional activity of AR. Therefore, the dual-luciferase assays through co-transfected with PSA luciferase (PSA-Luc) and Renilla plasmids were performed to evaluate the antiandrogen activity of the candidate compounds. As a result, both IMB-A6 (10 μM) and IMB-A8 (10 μM) significantly inhibited the AR transcriptional activity at the concentration of 10 μM in LNCaP cells (Figure 2C). However, in PC-3 cells with transferred exogenous AR gene, dual-luciferase assay of AR activity indicated that IMB-A6 (10 μM) had rather better inhibitory potency than IMB-A8 (10 μM). Thus we selected IMB-A6 as a potential antiandrogen for further mechanism studies.

### IMB-A6 Inhibits the Activity of Both WT and Mutated ARs

**FIGURE 3:**
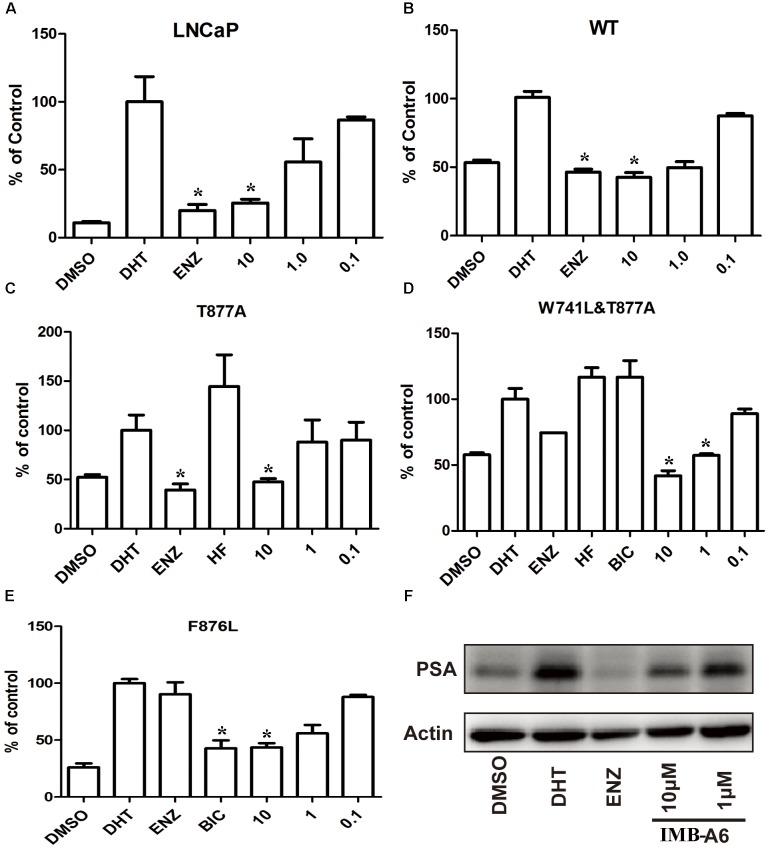
IMB-A6 is identified as a pan antiandrogen. **(A)** Effect of IMB-A6 on AR activity in LNCaP cells. **(B)** Effect of IMB-A6 on WT AR activity in PC-3 cells. **(C)** Effect of IMB-A6 on T877A AR activity in PC-3 cells. The T877A mutated AR was activated by hydroxyflutamide at 5 μM. **(D)** Effect of IMB-A6 on W741L + T877A AR activity in PC-3 cells. The T877A + W741C mutated AR was activated by both hydroxyflutamide and bicalutamide at 5 μM. **(E)** Effect of IMB-A6 on F876L AR activity in PC-3 cells. The F876L mutated AR was activated by enzalutamide at 5 μM. **(F)** Western blot analysis indicated that IMB-A6 suppressed DHT induced PSA protein expression in LNCaP cells at 1 and 10 μM. Plasmids expressing ARs were transiently transfected in PC-3 cells in dual luciferase assay. Experiments were in triplicate. ^∗^*P* < 0.05, ^∗∗^*P* < 0.01 vs. DHT group. BIC, bicalutamide; HF, hydroxyflutamide; ENZ, enzalutamide; DHT, dihydrotestosterone. All results are shown as mean ± s.d.

To further evaluate the antiandrogenic potency of IMB-A6, we investigated antiandrogenic activity of IMB-A6 against WT and T877A (FLU resistant mutant), F876L (ENZ and ARN509 resistant mutant), and W741C + T877A (FLU and BIC resistant mutant) mutated ARs in presence of DHT at 0.1 μM, 1 μM, and 10 μM. As a result, IMB-A6 inhibited the AR transcriptional activity in a dose-dependent manner in LNCaP cells (Figure 3A). In PC-3 cell with transferred exogenous AR genes, IMB-A6 demonstrated effective antiandrogenic activity in suppressing DHT-induced transcriptions of the WT, F876L, T877A, and W741C+T877A AR mutants in a dose-dependent manner (Figures 3B–E), which indicates IMB-A6 is a pan-antagonist against WT and mutated ARs. To further confirm the antiandrogenic activity of IMB-A6, we performed the western blot analysis to check its effect for the expression of PSA protein. As a result, IMB-A6 inhibited the DHT induced PSA expression in a dose-dependent manner at concentration of 1 μM, and 10 μM (Figure 3F). Thus, both dual-luciferase assay and western blot analysis indicated that IMB-A6 could efficiently inhibit the transcriptional activity of AR.

### IMB-A6 Inhibits AR Activity Through Binding to AF2 Site

**FIGURE 4:**
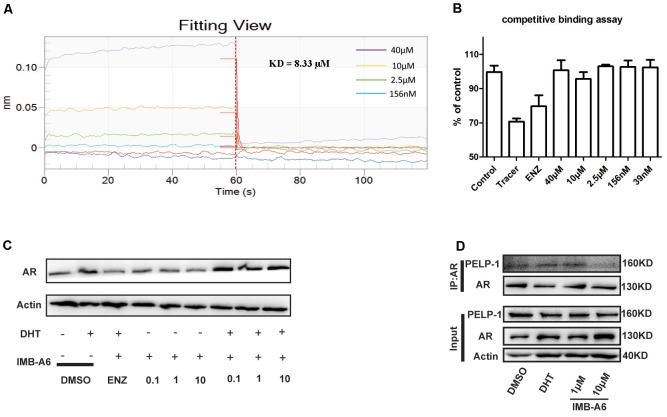
IMB-A6 inhibits AR activity through binding to AF2 site. **(A)** The Octet binding assay using biotinylated AR-LBD on superstreptavidin sensors and IMB-A6 indicated IMB-A6 bound directly to AR. **(B)** Competitive binding of IMB-A6 to the AR-LBD evaluated by AR fluorescence polarization (FP) assay showed that IMB-A6 did not bind to the HBP site. **(C)** Western blot analysis indicated that IMB-A6 did not suppress AR protein expression in LNCaP cells at 1 and 10 μM. **(D)** IMB-A6 significantly disrupted the interaction between AR and PELP-1 at 10 μM as a result of Co-immunoprecipitation assay.

We further investigated whether IMB-A6 inhibits AR activity through binding to AF2 site. Firstly, we performed the Octet binding assay using biotinylated AR-LBD on superstreptavidin sensors and IMB-A6 to test if IMB-A6 directly binds to AR-LBD. IMB-A6 had a dissociation equilibrium constant of 8.33 μM (Figure 4A), which demonstrated that IMB-A6 directly bound to AR-LBD. To verify if IMB-A6 bound to the site other than HBP, the AR fluorescence polarization (FP) assay was performed with PolarScreen AR competitor assay kit (P3018, Invitrogen). When the tracer is free in solution, its rotational mobility is greater than bound to the receptor, resulting in a low fluorescence polarization value. We have controlled the assay for minimal competition (DMSO vehicle), which has a maximum value of fluorescence polarization and for no receptor (tracer only), which represents the minimum value of the fluorescence polarization that can possibly be reached by a competitor. The ENZ was included as a positive control (Figure 4B). IMB-A6 did not reduce the fluorescence polarization value up to 40 μM, which indicated that IMB-A6 did not compete with the tracer in the HBP site and did not bind to the HBP site. Further western blot analysis indicated that IMB-A6 did not reduce the expression of AR both in absence or presence of DHT (Figure 4C), which indicated that IMB-A6 inhibited AR activity was not caused by the inhibition of AR expression. Proline-glutamic acid- and leucine-rich protein 1 (PELP1) is a co-regulator of AR, which binds at the AF2 site of AR, and plays a critical role in AR-mediated genomic signaling in PCa cells (Yang et al., 2012). Therefore, we investigated whether IMB-A6 interrupted the PELP1/AR interaction competing at AF2 site. Co-immunoprecipitation (Co-IP) studies of AR and PELP-1 were performed. As a result, IMB-A6 significantly disrupted the interaction between AR and PELP-1 at 10 μM (Figure 4D). All together, these evidences suggested that IMB-A6 inhibited AR activity through binding to the AF2 site.

### IMB-A6 Inhibits DHT Induced AR Nuclear Translocation

**FIGURE 5:**
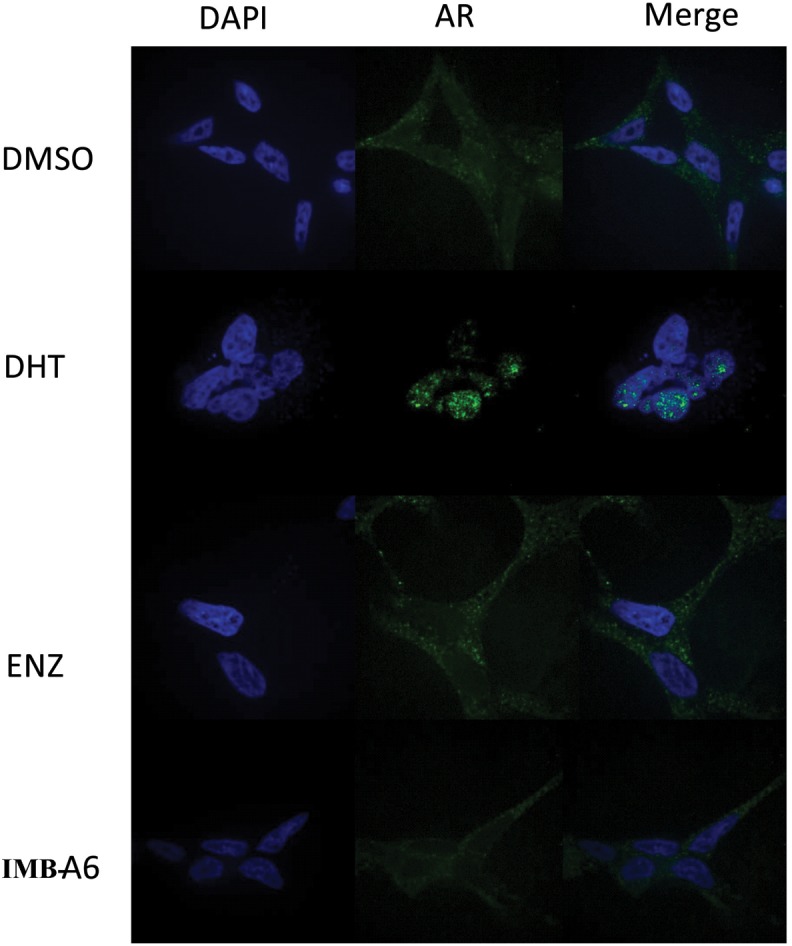
IMB-A6 efficiently inhibits DHT induced AR nuclear translocation. LNCaP cells were treated with DMSO, 1 nM DHT, 500 nM enzalutamide and 1 nM DHT, 5 μM IMB-A6 and 1 nM DHT in confocal assay. The counterstain DAPI was used to visualize cell nucleus.

Upon activation by androgens, AR translocates into the nucleus where it binds to androgen response element (ARE) of DNA. We then evaluated if IMB-A6 could affect DHT-induced translocation of AR from the cytoplasm to the nucleus using the confocal assay. The real-time nuclear localization of endogenous AR after 1 nM DHT treatment in the presence of 5 μM IMB-A6 or ENZ was evaluated. As a result, IMB-A6 blocked DHT-induced translocation of AR to the nucleus compared with the DHT-only treated group, which was comparable to ENZ or DHT-free (DMSO) group (Figure 5). The results indicated that IMB-A6 was able to inhibit DHT induced AR nuclear translocation and further confirmed that IMB-A6 was a bona fide antiandrogen.

### Identifying IMB-A6 Analogs as Antiandrogens Through Molecular Similarity

**FIGURE 6:**
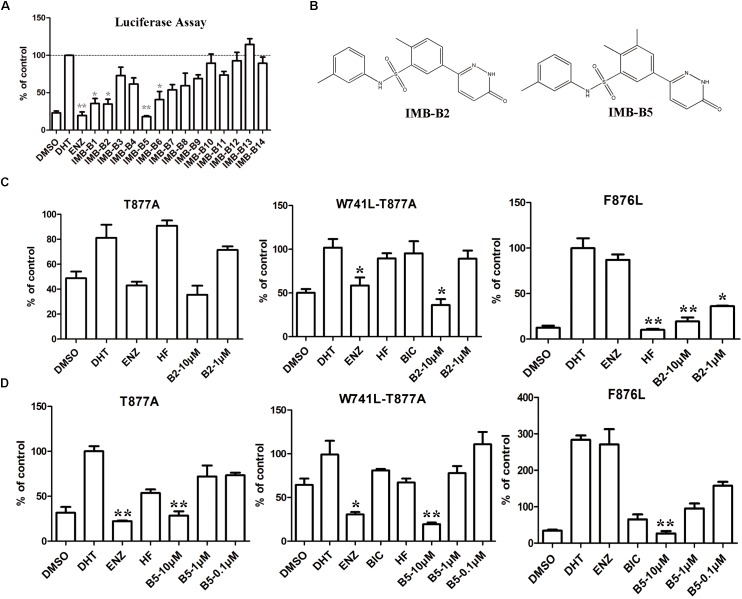
Identify IMB-A6 analogs as antiandrogens. **(A)** The dual-luciferase assay of AR activity in LNCaP cells for the analogs of IMB-A6. **(B)** The chemical structures of IMB-B2 and IMB-B5. **(C)** IMB-B2 demonstrated effective antiandrogenic activity in suppressing DHT-induced transcriptions of the F876L, T877A, and W741C+T877A AR mutants. **(D)** IMB-B5 demonstrated effective antiandrogenic activity in suppressing DHT-induced transcriptions of the F876L, T877A, and W741C+T877A AR mutants. Experiments were in triplicate. ^∗^*P* < 0.05, ^∗∗^*P* < 0.01 vs. DHT group. BIC, bicalutamide; HF, hydroxyflutamide; ENZ, enzalutamide; DHT, dihydrotestosterone. All results are shown as mean ± s.d.

To further obtain the active analogs of IMB-A6. Molecular similarity search based on the structure of IMB-A6 was performed (Tanimoto Coefficient > 70%) to furnish a new screening series of 248 compounds. These compounds were further screened through CDOCKER in DS and 14 compounds (IMB-B1-B14, Supplementary Table S3) were finally purchased for the further bioassay. As a result, the dual-luciferase assay of AR activity in LNCaP cells indicated that 8 of 14 compounds exhibited anti-androgenic activity at the concentration of 10 μM (Figure 6A). Among them, IMB-B2 and IMB-B5 (structures shown as Figure 6B) significantly inhibited the AR transcriptional activity. Next, we evaluated whether IMB-B2 and IMB-B5 inhibited the activity of mutated ARs. In PC-3 cells with transferred exogenous AR genes, IMB-B2 (Figure 6C) and IMB-B5 (Figure 6D) demonstrated effective antiandrogenic activity in suppressing DHT-induced transcriptions of the F876L, T877A, and W741C+T877A AR mutants in a dose-dependent manner. Therefore, the compounds that bearing IMB-A6 like scaffold represent a new type of antiandrogens.

The long terminal repeat (LTR) of mouse mammary tumor virus (MMTV) contains an androgen hormone response element. As we previously only used PSA reporter assay to evaluate the activity of AR, we preferred to use MMTV-luc reporter to confirm the antiandrogen activity of the active compounds (IMB-A6, IMB-B2, and IMB-B5). As results, the compounds IMB-A6, IMB-B2, and IMB-B5 efficiently inhibited the DHT induced MMTV level (Supplementary Figure S1), comparable to that of PSA, which indicated that the three compounds could efficiently inhibit the transcriptional activity of AR.

## Discussion

Structure-based drug design has become an efficient strategy in lead compound discovery. In this study, we first generated a pharmacophore model based on the alignment of the AR structures with the ligand binding to AF2 site. Through pharmacophore based screening, molecular docking, and subsequent bioassay, we identified IMB-A6 and its analogs as a new type of antiandrogen. Importantly, the binding assay indicated that IMB-A6 bound to AR-LBD but not to HBP site. Further Co-IP studies indicated that IMB-A6 could disrupt the interaction between AR and PELP-1 at the AF2 site, which suggested that IMB-A6 bound to the AF2 site to inhibit AR activity. However, such a hypothesis should be further confirmed by the structural biology methodology such as X-ray diffraction or NMR to verify the direct binding. IMB-A6 was able to inhibit the transcriptional activity of both WT-AR and mutated ARs and block the DHT-induced AR nuclear translocation. The IMB-A6 analogs obtained through molecular similarity search also indicated the pan-antagonistic activity against WT and mutated ARs. To date, there are several types of antiandrogens that bind to the AF2 site, including designed miniproteins, peptide mimics, and small molecules. The tanimoto similarity values between IMB-A6 and other AF2 binding small molecular compounds (Supplementary Table S4) were calculated through Open Babel were all below 0.25 (Supplementary Table S4), indicating that IMB-A6 represented a type of lead compound with a novel core-structure for further optimization. Our work thus provides another example for structural based lead compound discovery of AF2 binding ligand.

The AF2 site is essential for the recruitment of the various AR coactivators. The interactions between AR and cofactors at the AF2 site have been shown to be necessary in AR-mediated transcriptional regulation. Thus blocking interactions between AR and cofactors is an efficient way to develop the antiandrogen. Up to now, current clinically used antiandrogens targeting AR are all bound to the HBP of AR. The selective mutants in HBP site would lead to the resistance of PCa for current clinical antiandrogens, which might turn the antagonist to the agonist. As the AF2 site is formed via triggering agonist-induced conformational change upon the binding of androgen, the antiandrogen targeting the AF2 site should conquer the drug resistant mutants of the clinical antiandrogens. Herein, we found that IMB-A6 and its analogs could inhibit both WT and resistance mutated ARs. In addition, IMB-A6 could block DHT-induced nuclear translocation. Thus it suggests that antiandrogens targeting AF2 site represent a type of promising therapeutic agents for PCa. Importantly, other than PCa, the AF2 site may be a potential drug target site for the selective modulation of toxic AR activity leading to the disease like spinal bulbar muscular atrophy (SBMA). Two compounds, tolfenamic acid and 1-[2-(4-methylphenoxy)ethyl]-2-[(2-phenoxyethyl)sulfanyl]-1H-benzimidazole binding to AF2 site were found as top candidates for rescuing lethality, locomotor function and neuromuscular junction defects in SBMA flies (Badders et al., 2018).

Besides of AF2 site, the sites on AR other than HBP include BF3 site, DNA binding site, and AF1 site. To date, several ligands have been identified to bind to these sites and exhibiting potent activities in antagonizing the AR signaling. For examples, Li et al. (2014) found a small-molecule inhibitors selectively targeting the DNA-binding site, which could effectively inhibit the growth of ENZ-resistant PCa cells. Andersen et al. (2010) reported a small molecule EPI-001 from a library of marine sponge extracts which exerted high selective inhibition of AR NTD mediated activity through binding to AF1 site. Thus through targeting these sites, it provides alternative ways for developing the new type of antiandrogens in therapy of CRPC. The SBDD strategy in our work could also be suitable for developing the ligands targeting these sites.

Apart from the resistant mutations, other mechanisms were reported for the ENZ-resistance for CRPC. For instance, the expression of AR splice variants by CRPC like AR-V7 or AR-V567, which are missed the LBD domain, thus leading to the persistent AR signaling and resistance to ENZ therapies (Nakazawa et al., 2014). Also, the GR signaling activation is a general mechanism of resistance to antiandrogens. Accumulating data suggest that potent AR inhibition with ENZ can increase glucocorticoids receptor (GR) expression, which regulates about 50% of AR-responsive genes re-expression, thus promoting PCa progression despite the blockage of AR signaling (Narayanan et al., 2016). Another important resistance mechanism is the neuroendocrine differentiation in PCa, which leads to the treatment-induced AR independent PCa. Neuroendocrine PCa is resistant to antiandrogen therapy and usually associated with nodal or/and distant metastases (Kretschmer et al., 2015). As for the cases mentioned above, the antiandrogens targeting the AR-LBD including AF2 site, HBP site and BF3 site will not work anymore. Thus, there are still big challenges remaining in treatment of PCa.

## Author Contributions

YL, MW, and TW performed the experiments and analyzed the data. YX, XC, LH, and YH helped to set up the assay methodology. XL, ML, LH, and SC provided the resource and partial funding supports. JZ designed the experiments and wrote the original manuscript. JZ and SC reviewed and edited the manuscript.

## Conflict of Interest Statement

The authors declare that the research was conducted in the absence of any commercial or financial relationships that could be construed as a potential conflict of interest.

## Footnotes

1 www.pdb.org

2 www.fortebio.com
