# Supplementary material for: Structural Based Screening of Antiandrogen Targeting Activation Function-2 Binding Site
Source: Front Pharmacol. 2018 Nov 30;9:1419. doi: 10.3389/fphar.2018.01419 (PMC6284051; doi:10.3389/fphar.2018.01419)
Supplement: Supplementary file 1 [file Data_Sheet_1.docx]

**Structural based screening of antiandrogen targeting activation function-2 binding site**

Yangguang Liu^1, 2, &^, Meng Wu^1, 2&^, Tianqi Wang^2^, Yongli Xie^1,2^, Xiangling Cui^1, 2^, Liujun He^2^, Yang He^2^, Xiaoyu Li^2^,Mingliang Liu^2^, Laixing Hu^2*^, Shan Cen^2*^, Jinming Zhou^1,2 *^

^1^Key Laboratory of the Ministry of Education for Advanced Catalysis Materials, Department of Chemistry, Zhejiang Normal University, 688 Yingbin Road, Jinhua 321004, P. R. China.

^2^Institute of Medicinal Biotechnology, Chinese Academy of Medical Science, Beijing, China

**Table of content**

**Table S1** The structures of AR with small ligands binding to the AF2 site;

**Table S2.** The information of IMB-A Series;

**Table S3.** The information of IMB-B Series;

**Table S4.** The similarity analysis between IMB-A6 and other small molecular compounds targeting the AF2 site;

**Figure S1.** Effect of IMB-A6, IMB-B2 and IMB-B5 on wild-type AR activity in PC-3 cells respectively.

**Table S1** The structures of AR with small ligands binding to the AF2 site.

| PDB codes： | Ligands | PDB codes： | Ligands |
| --- | --- | --- | --- |
| 2PIO |  | 2YHD |  |
| 2PIQ |  | 2PIW |  |
| 2PIP |  | 2PIU |  |
| 2YLP |  | | |

**Table S2.** The information of IMB-A Series

| No. | ZINC IDs | MCULE IDs | Structure | Product URL |
| --- | --- | --- | --- | --- |
| A1 | ZINC55042618 | MCULE-1260679885 |  | <http://mcule.com/P-7810453/> |
| A2 | ZINC71752547 | MCULE-8340109341 |  | <http://mcule.com/P-9165129/> |
| A3 | ZINC71759540 | MCULE-1094579925 |  | <http://mcule.com/P-9286127/> |
| A4 | ZINC30690956 | MCULE-1022227849 |  | <http://mcule.com/P-32591192/> |
| A5 | ZINC13389058 | MCULE-3483000536 |  | <http://mcule.com/P-32534929/> |
| A6 | ZINC31534608 | MCULE-8289692294 |  | <http://mcule.com/P-32578292/> |
| A7 | ZINC69776841 | MCULE-8892470060 |  | <http://mcule.com/P-32789157/> |
| A8 | ZINC69664278 | MCULE-8219034897 |  | <http://mcule.com/P-21853191/> |
| A9 | ZINC58417914 | MCULE-2578977380 |  | <http://mcule.com/P-4230739/> |
| A10 | ZINC58007747 | MCULE-5443383565 |  | <http://mcule.com/P-3746716/> |
| A11 | ZINC35287343 | MCULE-4827623689 |  | <http://mcule.com/P-19569330/> |
| A12 | ZINC20602734 | MCULE-6084259220 |  | <http://mcule.com/P-24001549/> |

**Table S3.** The information of IMB-B Series

| NO. | ZINC ID | MCULE ID | Structure | Product URL |
| --- | --- | --- | --- | --- |
| B1 | ZINC15669293 | MCULE-9023052687 |  | <http://mcule.com/P-6448341/> |
| B2 | ZINC15669334 | MCULE-2326813695 |  | <http://mcule.com/P-6448673/> |
| B3 | ZINC20991888 | MCULE-7293567201 |  | <http://mcule.com/P-8567207/> |
| B4 | ZINC21917494 | MCULE-4354932915 |  | <http://mcule.com/P-8567273/> |
| B5 | ZINC15866609 | MCULE-9906433448 |  | <http://mcule.com/P-9621554/> |
| B6 | ZINC15669324 | MCULE-8044729119 |  | <http://mcule.com/P-6448604/> |
| B7 | ZINC57819873 | MCULE-1966967493 |  | <http://mcule.com/P-32724962/> |
| B8 | ZINC09562876 | MCULE-8860482182 |  | <http://mcule.com/P-7924004/> |
| B9 | ZINC57819992 | MCULE-8075094519 |  | <http://mcule.com/P-32724965/> |
| B10 | ZINC57820011 | MCULE-7774413791 |  | <http://mcule.com/P-32724966/> |
| B11 | ZINC10514307 | MCULE-5937832847 |  | <http://mcule.com/P-10981898/> |
| B12 | ZINC79107651 | MCULE-4158478868 |  | <http://mcule.com/P-21978357/> |
| B13 | ZINC31776613 | MCULE-7903427104 |  | <http://mcule.com/P-11594294/> |
| B14 | ZINC06551887 | MCULE-6039947120 |  | <http://mcule.com/P-32048054/> |

**Table S4.** The similarity analysis between IMB-A6 and other small molecular compounds targeting the AF2 site. The similarity was calculated using Open Babel.

| **Compounds** | **Structure** | **Timinoto Score** |
| --- | --- | --- |
| Fragment1 |  | 0.18 |
| Fragment2 |  | 0.24 |
| Fragment3 |  | 0.19 |
| Fragment4 |  | 0.24 |
| Fragment5 |  | 0.18 |
| Fragment6 |  | 0.20 |
| Ligand1 |  | 0.20 |
| Ligand2 |  | 0.20 |
| Ligand3 |  | 0.20 |
| Ligand4 |  | 0.19 |
| Ligand5 |  | 0.17 |
| Ligand6 |  | 0.18 |
| Ligand7 |  | 0.16 |


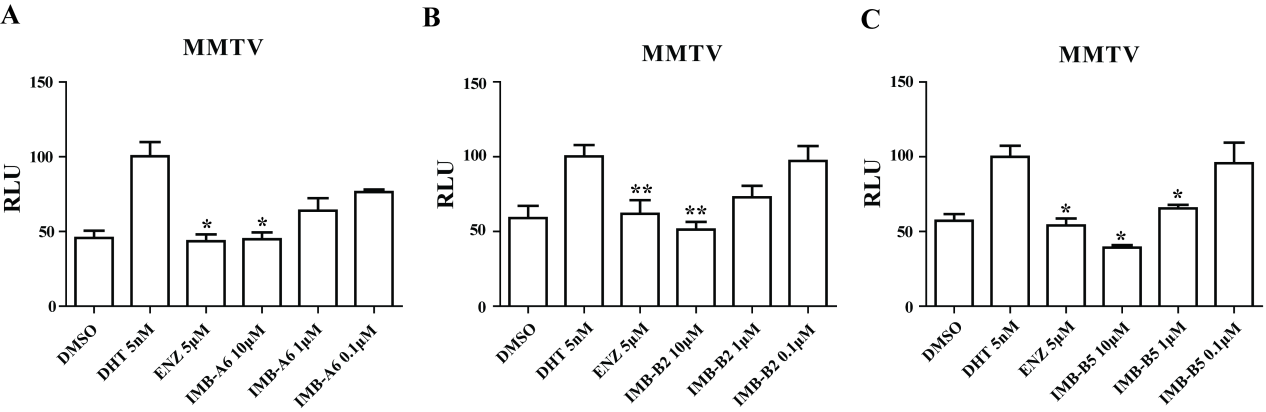
**Figure S1.** Effect of IMB-A6 (A), IMB-B2 (B) and IMB-B5 (C) on wild-type AR activity in PC-3 cells respectively. The MMTV-Luc was as the reporters. Plasmid expressing AR was transiently transfected in PC-3 cells in dual luciferase assay. Experiments were in triplicate. *P < 0.05, **P < 0.01 vs DHT group. ENZ: enzalutamide; DHT: dihydrotestosterone. All results are shown as mean ± s.d.
